# Gadolinium Complex with Tris-Hydroxypyridinone as an Input for New Imaging Probes: Thermodynamic Stability, Molecular Modeling and Biodistribution

**DOI:** 10.3390/molecules30061295

**Published:** 2025-03-13

**Authors:** Inês Dias, Lurdes Gano, Sílvia Chaves, M. Amélia Santos

**Affiliations:** 1 Centro de Química Estrutural, Institute of Molecular Sciences, Departamento de Engenharia Química, Instituto Superior Técnico, Universidade de Lisboa, Av. Rovisco Pais 1, 1049-001 Lisboa, Portugal; 2 Centro de Ciências e Tecnologias Nucleares, Instituto Superior Técnico, Universidade de Lisboa, Estrada Nacional 10 (km 139,7), Loures, 2695-066 Bobadela, Portugal; lgano@ctn.tecnico.ulisboa.pt

**Keywords:** hydroxypyridinones, MRI contrast agents, gadolinium complex, thermodynamic stability, biodistribution

## Abstract

The development of gadolinium-based magnetic resonance imaging (MRI) contrast agents (CAs) is a highly challenging and demanding research field in metal-coordination medicinal chemistry. The recognized high capacity of hydroxypyridinone (HOPO)-based compounds to coordinate Gd (III) led us to evaluate the set of physic–chemical–biological properties of a new Gd (III) complex with a hexadentate tripodal ligand (H_3_L) containing three 3,4-HOPO chelating moieties attached to an anchoring cyclohexane backbone. In particular, the thermodynamic stability constants of the complex were evaluated by potentiometry, showing the formation of a highly stable (1:1) Gd-L complex (log *β*_GdL_ = 26.59), with full coordination even in an acid-neutral pH under the experimental conditions used. Molecular simulations of the Gd (III) complex revealed a minimum energy structure with somewhat-distorted octahedral geometry, involving full metal hexa-coordination by the three bidentate moieties of the ligand arms, indicating that an extra water molecule should be coordinated to the metal ion, an important feature for the CAs (and the required enhancement of water proton relaxivity). In vivo biodistribution studies with the ^67^Ga complex, as a surrogate of the corresponding Gd complex, showed in vivo stability and rapid excretion from the animal body. Though deserving further investigation, these results may give an input on future perspectives towards new MRI diagnostic agents.

## 1. Introduction

Paramagnetic metal ion complexes, mostly based on gadolinium (Gd(III)), have been investigated and used over the last three decades as magnetic resonance imaging (MRI) contrast agents (CAs) [1,2]. Gadolinium-based contrast agents (GBCAs) are now employed routinely to enhance the sensitivity and specificity of MRI examinations. The commercially available GBCAs are Gd(III) complexes with polyaminopolycarboxylate ligands enabling octadentate coordination to the metal ion and can be classified into two groups according to the ligand structure: macrocyclic (e.g., Dotarem) or linear (e.g., Omniscan).

One of the major factors that control the efficiency of MRI CAs is their relaxivity, which must be improved in order to reduce the amount of CA required. The relaxivity is a measure of the CA sensitivity. It reflects the relaxation rate of surrounding water protons, being namely determined by exchange rates between water molecules in the inner- and the outer-coordination sphere of the metal ion. This includes the water of the tissues and organs, though other parameters can affect the relaxivity of GBCAs, such as the structure and molecular weight of the ligand, as well as their rotational correlation time [3].

Despite all the advantages of GBCAs, it is also crucial to take into consideration that gadolinium is very toxic as a free ion, due to its recent association with nephrogenic systemic fibrosis (a rare but potentially fatal outcome for some patients with renal failure) [4,5]. Thus, for clinical applications, a key aspect of the Gd(III) complexes used as CAs must be their high thermodynamic stability [6] as well as kinetic inertness. These properties are intended to guarantee the long-term resistance of the GBCAs to Gd(III) dissociation (and the release of the toxic metal ion), for which the respective dissociation rate should be slower than the elimination rate in the patient body [7].

On the other hand, since the trivalent gadolinium prefers a coordination number of 9, its octadentate coordination with the currently available GBCAs, based on chelates with polyaminoacid ligands, leaves only one coordination site free for an inner sphere water molecule. Therefore, to improve the relaxivity by raising the hydration state of the contrast, the strategy of reducing the number of coordination sites provided by the ligands can be adopted, though for this family of Gd chelates it would imply both a reduction in the thermodynamic stability of the GBCAs as well as raised toxicity concerns. Aimed at overcoming this problem, a new group of alternative Gd chelates were developed in 2006 by the group of K.N. Raymond [8], and in this line, a series of tripodal ligands containing three hydroxypyridinone (HOPO) chelating moieties, such as TREN-1-Me-3,2-HOPO, has been extensively investigated. The Gd(III) complexes with these ligands revealed to contain two or three water molecules coordinated to the metal ion, which conferred remarkable high relaxation efficiencies, besides high thermodynamic stability (log *K*_GdL_ = 19.2) [8,9].

**Scheme 1:**
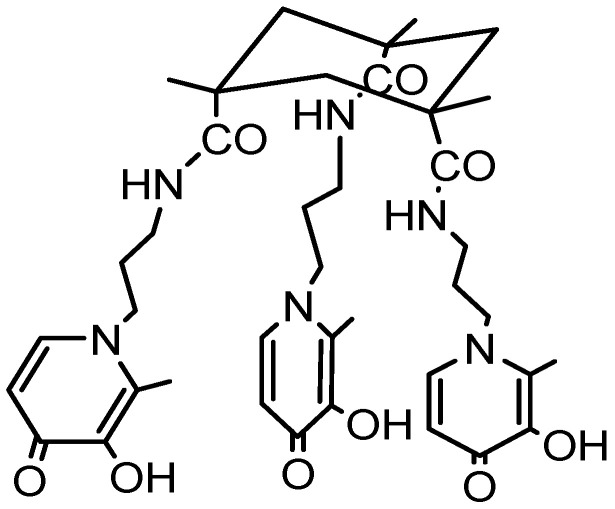
Structural formula of the hexadentate tripodal compound KEMPPr (3,4-HP)_3_.

Following an identical strategy, different tripodal tris-3-hydroxy-4-pyridinone (tris-3,4-HOPO) ligands were recently developed and evaluated for the magnetic and complexation properties of the corresponding Gd complexes [10,11]. The obtained results confirmed the capacity of these ligands to form very stable Gd complexes (log *K*_GdHL_ = 26.35 [10], log *K*_GdL_ = 21.22 [11]) with hexadentate coordination and two free sites left for water coordination to the metal ions to enable the efficient paramagnetic enhancement of water proton relaxation. Inspired by these findings, we have decided to investigate the properties of a Gd(III) complex with another tripodal tris-3,4-HOPO compound, KEMPPr(3,4-HP)_3_ (see Scheme 1), with three 3,4-HOPO chelating moieties attached to *cis*,*cis*-1,3,5-trimethylcyclohexane–1,3,5-tricarboxylic acid (Kemp’s triacid). This ligand has previously revealed a high capacity to wrap and sequestrate three positive *hard* metal ions (Fe(III), Ga(III), Al(III)) through hexa-coordination of the 1:1 M/L complexes [11]. In particular, described herein are the results of the studies on the chelating ability of KEMPPr(3,4-HP)_3_ towards Gd (III), namely its solution equilibrium thermodynamic stability as a measure of its capacity to form strong hexa-coordinated 1:1 Gd/L complexes, and to potentially leave extra positions for the coordination of water molecules to Gd (III). Furthermore, molecular modeling calculations, based on density functional theory (DFT), were also performed to predict the probable metal complex structure and also the number of water molecules coordinated to the Gd complex. In silico evaluation was also performed to obtain insight on some of the pharmacokinetic parameters of the metal complex. Finally, studies in mice were presented to assess the in vivo stability and biodistribution profile of the ^67^Ga complex as a surrogate model of the analogue Gd complex.

## 2. Results and Discussion

### 2.1. Gd Chelation Studies

The determination of the thermodynamic stability constants of the Gd(III) complex required a previous evaluation of the ligand protonation constants to be subsequently introduced in the Gd(III) complexation model. The acid-base behavior of KEMPPr(3,4-HP)_3_ was already studied in aqueous medium [12], revealing that although the compound was isolated in the neutral three-protonated form, H_3_L, its fully protonated species (H_6_L^3+^) has six dissociable protons. The three first protonation constants correspond to the phenolic groups of the 3,4-HP moieties, while the last three are ascribed to the pyridyl nitrogen atoms. Although this compound is water soluble, previously performed metal complexation studies revealed some solubility problems, namely due to the formation of the neutral M(III)-L complexes as well as the possibility of precipitation due to metal hydrolysis. In fact, in the formerly reported iron complexation studies with KEMPPr(3,4-HP)_3_, a mixed medium (methanol/water) was also used, besides aqueous medium, to try to avoid these problems [12]. Since DMSO–water solvents are widely used to enhance solubilities both in chemical and biological systems, it was decided herein to use a unique mixed DMSO/water medium (50%, *w*/*w*), and so the protonation constants had to be re-calculated under these experimental conditions in order to introduce the corresponding acid–base data in the Gd(III) complexation model. Even though under the potentiometric experimental conditions used herein (*C*_L_ = 3.2 × 10^−4^ M) some solubility problems can arise in the aqueous medium, in cell biological assays, the concentration of ligand used is lower (<7 μM) and thus the final DMSO concentration in culture media would be even lower (<1%); consequently, this was not predicted to induce damages in the biological tissues.

**Figure 1:**
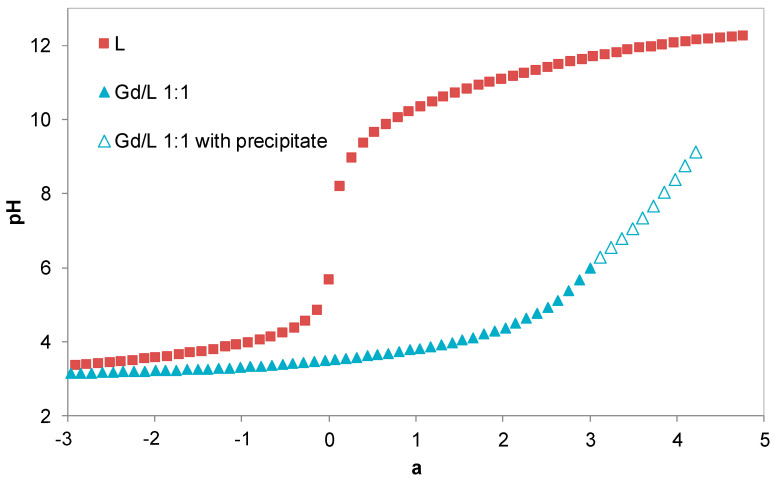
Potentiometric titration curves for the compound (H_3_L), KEMPPr(3,4-HP)_3_ and the system Gd(III)/L 1:1 (*C_L_*= 3.2 × 10^−4^ M, 50% *w*/*w* DMSO/water medium). **a** represents the moles of the added base per mole of ligand. The empty symbols stand for experimental points that were not included in the calculations.

All the studies were performed by potentiometric titrations of the ligand alone or in the presence of a 1:1 Gd/L stoichiometric ratio (Figure 1), which allowed for the determination of the protonation constants of KEMPPr(3,4-HP)_3_, as well as the stability constants of the formed Gd(III) complexes, from the best fitting of the experimental curves with the Hyperquad program [13] (Table 1). Other Gd-to-ligand molar ratios were not considered in the present study, because it was admitted that species with a 1:2 stoichiometric ratio are present only at quite low concentrations for pH ca 7 [10].

When using this mixed aqueous–DMSO medium, operational pH values are obtained, for which the proton activity differs from that for aqueous medium due to differences in standard-state chemical potentials, and so a different pH scale is obtained [14]. Therefore, only cautious comparisons between values obtained in DMSO–water and aqueous media are possible. Notwithstanding the differences between the working media, the values obtained herein for the fourth, fifth and sixth protonation constants of KEMPPr(3,4-HP)_3_ (log *K*_i_ = 3.04–3.96) are similar to those previously reported in aqueous medium for this and other tripodal compounds [11,12,15]. However, slightly higher values are found for the first three values of the protonation constants, in accordance with previous studies on the influence of the composition of DMSO/water media on the acid–base equilibrium that referred to the importance of the electrostatic forces [16,17]. Therefore, the basicity of negatively charged O-sites (from the HP moieties) is expected to increase with the lower hydrating capacity of the 50% *w*/*w* DMSO/water medium. Since the Gibbs energy of proton transfer from water to the 50% *w*/*w* DMSO/water is quite negative (−5.11 kcal/mol) [18], this means that this mixed solvent has a higher solvating capacity, with DMSO forming stronger electrostatic and covalent bonds than water. In fact, the oxygen in DMSO is in the borderline between *hard* and *soft* donors, which gives it a high possibility of participating in covalent bonding.

Since the protonation centers contained in one arm of the tripodal ligand are quite far away from the similar ones in the remaining two arms, they can be considered equivalent basic groups, for which a difference in the log *K* values of 0.602 could be expected [19]. In fact, a somehow higher difference (1.1) is found herein between log *K*_2_ and log *K*_3_, being otherwise similar to a previously reported value (1.15 [11]) between log *K*_4_ and log *K*_5_ for H_3_L2 (Table 1), which can eventually result from the establishment of some hydrogen bond interactions and concomitant minor inequivalence between the arm protonation centers.

**Figure 2:**
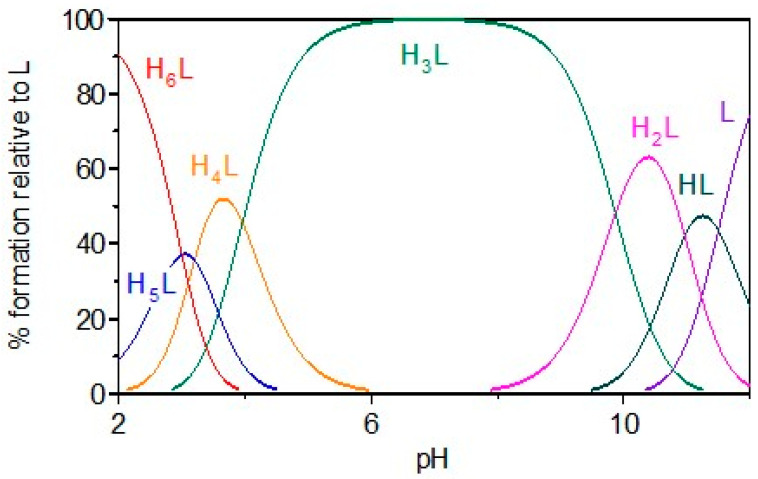
Species distribution curves of KEMPPr(3,4-HP)_3_ (*C*_L_ = 3.2 × 10^−4^ M, 50% (*w*/*w*) DMSO/water medium).

The species distribution plot in Figure 2 shows the predominance of the neutral H_3_L species in the pH range 3.4–9.9, which means that at a physiological pH of (7.4), the composition of the solution is 99.95% H_3_L and only 0.05% monocharged H_4_L^+^ species.

**Table 1:** Stepwise protonation constants (log *K*_i_) ^a^, global formation constants ^b^ of the Gd (III) complexes and pGd ^c^ (50% *w*/*w* DMSO/water, *T* = 25.0 ± 0.1 °C, *I* = 0.1 M KCl), as well as comparison values for some tripodal compounds.

Compound	log *K*_i_	(m,h,l)	log *β* (Gd_m_H_h_L_l_)
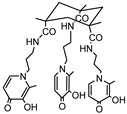 **KEMPPr(3,4-HP)_3_**	11.5(4) 10.97(5) 9.87(6) 3.96(7) 3.19(8) 3.04(9)	(1,4,1) (1,2,1) (1,1,1) (1,0,1) **pGd**	41.35(8) 35.17(7) 31.30(4) 26.59(8) 13.2 ^d^
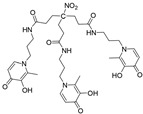 **H_3_L2** ^e^	9.93(2) 9.75(4) 9.18(5) 4.26(5) 3.11(6) 2.77(7)	(1,4,1) (1,2,1) (1,0,1) **pGd**	37.74(4) 30.03(6) 21.22(5) 14.3
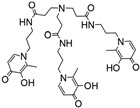 **NTP(PrHP)_3_**	9.95 ^f^ 9.84 ^f^ 9.09 ^f^ 6.77 ^f^ 3.81 ^f^ 3.14 ^f^ 2.76 ^f^	(1,5,1) (1,4,1) (1,3,1) (1,2,1) (1,1,1) (1,5,2) (1,3,2) **pGd**	42.8 ^g^ 39.46 ^g^ 35.69 ^g^ 31.16 ^g^ 26.35 ^g^ 65.3 ^g^ 52.3 ^g^ 12.3

^a^ *K*_i_ = [H_i_L]/[H_i-1_L][H]. ^b^ *β* (Gd_m_H_h_L) = [Gd_m_H_h_L_l_]/[Gd]^m^[H]^h^[L]^l^. ^c^ pGd value at pH = 7 (pGd = −log [Gd(III)], *C*_L_ = 10^−5^ M, *C*_L_/*C*_Gd_ = 10). ^d^ pGd at pH = 6. ^e^ in water [11]. ^f^ in water [15]. ^g^ in water [10].

**Figure 3:**
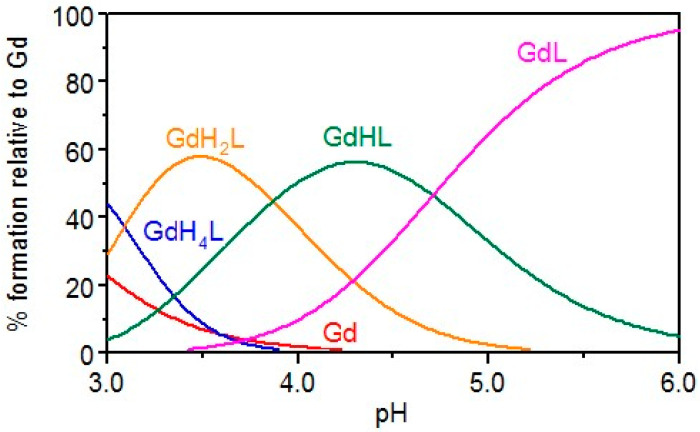
Species distribution curves for the Gd (III)/KEMPPr(3,4-HP)_3_ 1:1 system (*C*_L_ = 3.2 × 10^−4^ M, 50% *w*/*w* DMSO/water medium).

Analysis of the potentiometric titration curves (Figure 1), namely those obtained for the Gd(III)/KEMPPr(3,4-HP)_3_ system under 1:1 (Gd/L) stoichiometry, evidences a change in the deprotonation profile of the ligand due to its coordination to Gd (III). Since some precipitation was detected above pH = 6, the corresponding experimental points were not used in the calculations to obtain the Gd (III) complexation model. The obtained Gd(III) complexation model presents some similarities to that previously found for H_3_L_2_ (see Table 1) [11], firstly involving the formation of a bis-chelated GdH_4_L^4+^ species, a GdH_2_L^2+^ tetra-chelated complex, and afterwards the formation of a neutral hexa-coordinated GdL species. In fact, the analysis of the species distribution curves contained in Figure 3 shows that, under the used experimental conditions (50% *w*/*w* DMSO/water, C_L_ = C_Ga_ = 3.2 × 10^−4^ M), the Gd(III) complexation begins at pH ca 2, the bis-chelated complex is not very stable when compared with GdH_2_L^2+^ or GdL and the neutral GdL complex begins to form at pH ca 3.5, and becomes prevalent at pH 4.8. Concerning the Gd(III)/H_3_L2 system (in water, C_L_ = C_Gd_ = 3.2 × 10^−4^ M), the Gd(III) complexation also begins at pH ca 2, and the GdL species forms at pH ca 3.7 and becomes dominant at pH 4.5 [11]. For the Gd (III)/NTP (PrHP)_3_ system (in water), the GdL species is not involved in the proposed model [10].

The pGd value (pGd = −log [Gd (III)], *C*_L_ = 10^−5^ M, *C*_L_/*C*_Gd_ = 10) [20] presented in Table 1 for the Gd(III)/KEMPPr(3,4-HP)_3_ system is calculated at pH 6, while those for the comparison compounds are at pH = 7. Besides differences in the working media and pH, the pGd values obtained for KEMPPr(3,4-HP)_3_ and H_3_L2 show a somewhat higher chelating capacity towards Gd(III) (pGd = 13.2–14.3) than NTP(PrHP)_3_ (pGd = 12.3). Therefore, it seems that the higher backbone rigidity and shorter arms of KEMPPr(3,4-HP)_3_, when compared with H_3_L2 or NTP(PrHP)_3_, do not interfere with the Gd-chelating ability of these tripodal 3,4-HP derivatives. In fact, there are other tripodal compounds that are stronger Gd chelators (pGd ca 19–20 at pH 7.4), which may be attributed to a better spatial arrangement in the coordination core around the metal ion, e.g., with 3,2-HOPO or pyrone moieties, as well as the standard Gd (III) ligands, diethylenetriamine penta-acetic acid (DTPA, pGd = 19.1) and 1,4,7,10-tetraazacyclododecane *N*,*N*′,*N*″,*N*‴-tetra-acetic acid (DOTA, pGd = 20.4) are at pH 7.4 [21,22,23]. For these last two cases (DOTA and DTPA), there are higher coordination numbers for their Gd complexes when compared with that of KEMPPr(3,4-HP)_3_, and the similarity of their pGd values with 3,2-HOPO tripodal derivatives can be mainly attributed to the higher chelating strength of the 3,2-HOPO unities compared with that of carboxylic units, as well as a better pre-organization of these moieties for metal coordination.

Finally, the solution equilibrium studies performed herein show that KEMPPr(3,4-HP)_3_ is able to form a stable hexa-coordinated GdL complex, although the obtained equilibrium model is related to the experimental conditions undertaken, and so in vivo extrapolations must be made with care.

### 2.2. Molecular Modeling of the Gd Complex

In order to obtain some insight into the molecular structure of the Gd complex and to evaluate the possibility of enclosing water molecules in the Gd coordination shell, a prominent feature for its magnetic properties, DFT calculations were performed. Molecular simulations were obtained with full geometry optimization of the gadolinium complexes (with one or two water molecules coordinated to Gd) by quantum mechanical calculations based on the DFT methods included in the Gaussian 03 software [24,25], with the B3LYP hybrid functional [26] and the Stuttgart/Dresden ECP (SDD) basis set [27,28]. No symmetry constraints were considered during geometry optimization in vacuum.

**Figure 4:**
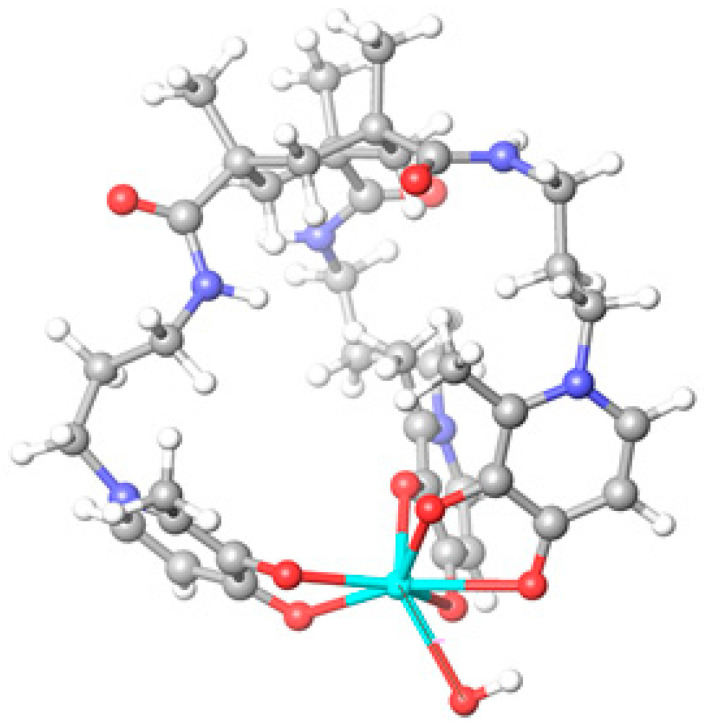
DFT-minimized structure of the Gd-KEMPPr(3,4-HP)_3_·1H_2_O complex, containing one water molecule in the coordination shell. Coloring of atoms: Gd (light blue), N (blue), O (red), C (gray) and H (white).

These calculations allow us to conclude that the Gd-KEMPPr(3,4-HP)_3_ complex should present a distorted octahedral geometry with hexacoordination provided by the three bidentate 3,4-HOPO moieties of the ligand to Gd(III). The energy-minimized structure obtained for the 1:1 Gd complex with one coordinated water molecule is shown in Figure 4. Analysis of the model complex shows that the backbone cyclohexane ring (chair conformation) is in the opposite direction relative to the coordination core, seeming to be responsible by a certain degree of distortion of the Gd coordination geometry, which may also make coordination with another water molecule difficult.

The coordination bond distances obtained for the Gd-KEMPPr(3,4-HP)_3_.1H_2_O complex are 2.41(9) Å for Gd-O_ligand_ (average value and standard deviation) and 2.524 Å for Gd-O_water_. These calculated distances for the Gd-O_ligand_ bonds agree well with the previous results reported for GdL1.2H_2_O (2.42(8) Å) [10], GdL2.1H_2_O (2.39(9) Å) [11], GdL2.2H_2_O (2.40(6) Å) [11] and the X-ray structure of the complex Gd(TREN-Me-3,2-HOPO)·2H_2_O (average 2.38(3) Å) [21]. The Gd-O_water_ bond length (2.524 Å) determined herein is also similar to other already published bond lengths: GdL1.2H_2_O (average 2.58(7) Å) [10], GdL2.1H_2_O (2.434 Å) [11] and GdL2.2H_2_O (2.436 and 2.537 Å) [11], with both ligands L1 and L2 presenting longer arms than KEMPPr(3,4-HP)_3_ (seven against against atoms); the values found for the Gd-O_water_ bond lengths in the X-ray structures of Gd(TREN-Me-3,2-HOPO)·2H_2_O (2.436 and 2.446 Å) [21] (ligand with four atom-length arms) and for the nine-coordinate complex [Gd(DTPA-BEA).H_2_O] (2.423 Å) [29] are also under the same order of magnitude.

The Gd-O_ligand_ (average 2.36(4) Å and 2.38(5) Å) and Gd-O_water_ (average 2.45(2) Å and 2.46(1) Å) bond distances found in the X-ray crystal structures of two acetylacetonate complexes (Gd(pta)_3_.2H_2_O and Gd(bfa)_3_.2H_2_O) are also similar to those calculated herein for Gd-KEMPPr(3,4-HP)_3_·1H_2_O, although those ligands are bidentate and not tripodal [30].

In conclusion, even though two water molecules could be accepted to coordinate to the metal ion, these calculations suggested that a minimum energy complex structure only allowed the extra-coordination of one water molecule to Gd. Nevertheless, the actual number of water molecules in the inner coordination sphere can only be confirmed by the experimental assessment of the relaxometric properties of the Gd(III) complex by ^17^O nuclear magnetic resonance (^17^O NMR) and nuclear magnetic relaxation dispersion (^1^H NMRD). If the actual number of water molecules in the Gd coordination sphere is one instead of two, this could result in lower relaxivity and sensitivity, possibly requiring an increase in the magnetic field strength for the contrast enhancement characteristics.

### 2.3. In Silico Prediction of ADME Properties and Drug Likeness

The molecular properties and pharmacokinetic parameters of the Gd-KEMPPr(3,4-HP)_3_ are determined using the online SwissADME platform (http://www.swissadme.ch, accessed on the 3 May 2025). They include physicochemical properties, such as molecular weight (MW) = 905.11 g/mol), number of hydrogen bond donors (HBH = 3), number of hydrogen bond acceptors (HBA = 12), number of rotatable bonds = 0, topological polar surface area (TPSA = 180.99 Ӑ^2^), lipophilicity, consensus value (average of all five predictions) log P_o/w_ = 0.39, water solubility, log *S* (ESOL = −6.48; SILICOS-IT = −4.43) mg/mL (moderately soluble), pharmacokinetics (low gastrointestinal absorption GI and “no” BBB permeant (according to the white of a boiled egg)). Therefore, the drug likeness of the complex appears limited for oral bioavailability, namely because of Lipinski’s rule (two violations: MW > 500, HBA > 10) [31] and the Veber (GSK) filter (one violation: TPSA > 140) [32].

The suitable physicochemical space for oral bioavailability is schematically represented in the colored zone of Figure S1, which includes a range of six parameters (lipophilicity, MW, polarity (TPSA), insolubility, insaturation, flexibility). Considering the results obtained from the SwissADME platform, the transport of this Gd complex across the blood–brain barrier appears compromised. Besides the moderate water solubility, the striking parameters for the oral bioavailability are the high TPSA and high MW, which typically contribute to reduced BBB permeability. Although, the lipophilicity herein does not appear to be a relevant factor for unfavorable barrier to BBB crossing, a variety of statistical techniques have suggested that lipophilicity and molecular weight are the most important factors in determining the permeability of drug candidates [33]. It is worth noting that, while these in silico predictions provide valuable insights, further experimental validation using methods such as the PAMPA-BBB assay could be used to confirm the actual BBB permeability of this complex [34]. Future studies could also be considered for improvements in BBB permeability to ensure that this Gd(III) chelate can effectively reach its main biotargets, namely with the use of nanoparticles or liposomes [3,35,36].

### 2.4. Biodistribution of the Gd-KEMPPr(3,4-HP)_3_ Complex

**Figure 5:**
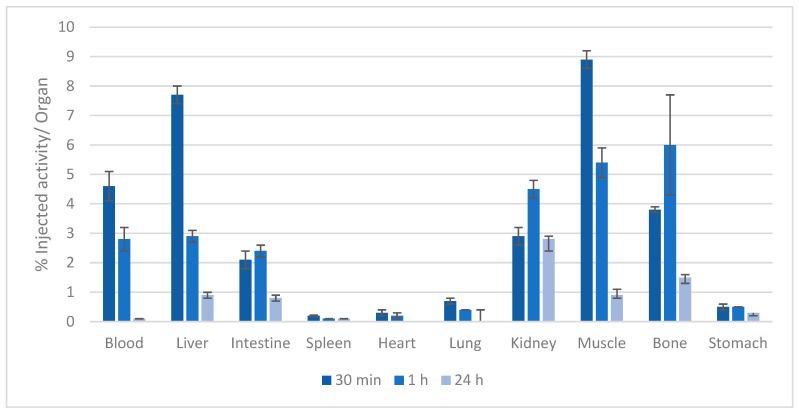
Biodistribution of ^67^Ga-KEMPPr(3,4-HP)_3_ in CD1 mice at 30 min, 1 h and 24 h after administration.

**Figure 6:**
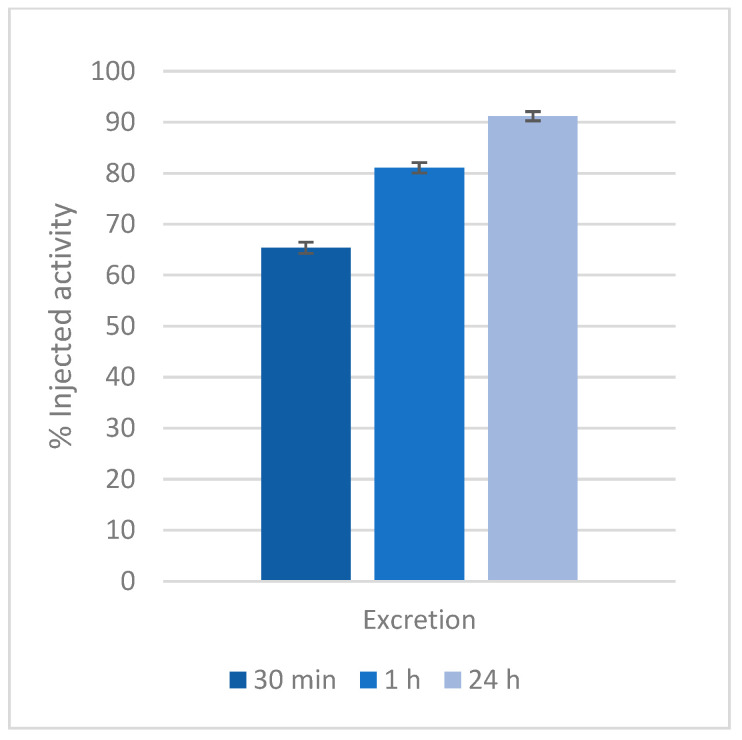
Total excretion of ^67^Ga-KEMPPr(3,4-HP)_3_ from whole animal body at 30 min, 1 h and 24 h after administration in CD1 mice.

In the absence of lab facilities to evaluate the in vivo MRI properties and biodistribution of the Gd-KEMPPr(3,4-HP)_3_ complex [37], the biodistribution and excretion profiles of the analogue ^67^Ga-KEMPPr (3,4-HP)_3_ complex, as a surrogate of the Gd complex, are evaluated in CD-1 mice at 30 min, 1 h and 24 h after the administration of the radiotracer (Figure 5 and Figure 6), similarly to the methodology previously reported by us [12]. The use of Ga-67 complex as a surrogate of the Gd complex can raise some questions, due to their different positions in the periodic table of the elements and different magnetic properties (diamagnetic and ferromagnetic, respectively). Gadolinium-based contrast agents (GBCAs) are commonly used in clinical magnetic resonance imaging (MRI), while the radioisotopes of gallium, Ga-67 and Ga-68, are mainly used in nuclear medicine as imaging agents. Nevertheless, we have decided to carry out this study considering the importance of the biodistribution and clearance profiles of Gd contrast agents to assess their potential as imaging probes, especially due to the known risk of nephrogenic systemic fibrosis (NSF) associated with GBCAs [4]. Similar approaches have been recently reported [38], in which a complex of DTPA with ^86^Y, a radionuclide used for positron emission tomography (PET), was used to evaluate the biodistribution and clearance in rats as a surrogate of the corresponding Gd complex, otherwise with prospects for multimodality molecular imaging.

Data from these studies demonstrate a fast blood clearance and a rapid washout from the main organs and tissues, since the % I.A./organ is lower than 1% for all major organs, except for the kidney, at 24 h post-injection (p.i.). The highest uptake in the kidneys and the high excretion rate of radioactivity from the whole animal body (65.4 ± 1.1, 81.1 ± 0.1 and 91.2 ± 0.9% I.A. at 30 min, 1 h and 24 h, respectively) indicate that the complex is predominantly eliminated by the urinary pathway.

The results of these studies suggest a high in vivo stability of the ^67^Ga-KEMPPr(3,4-HP)_3_ complex, as the biodistribution pattern related to the main radiochemical impurity species (^67^Ga hydroxides Ga(OH)_3_ and Ga(OH)_4_^−^ and free ^67^Ga^3+^) is well established and was not found.

Overall, these in vivo studies show a favorable biodistribution profile of this ^67^Ga-KEMPPr(3,4-HP)_3_ complex, with no relevant uptake in any organ except the excretion path at 24 h p.i., a high in vivo stability and the rapid excretion from the animal body.

## 3. Materials and Methods

### 3.1. Gd Complexation Studies

#### 3.1.1. Materials and Equipment

The compound KEMPPr(3,4-HP)_3_ was synthesized according to the previously published procedure [12]. The GdCl_3_ stock solution (9.8 × 10^−3^ M) was prepared from the gadolinium salt in HCl 0.1 M medium to avoid hydrolysis, and standardized by inductively coupled plasma emission (ICP). The 0.1 M HCl solution used in the calibration of the glass electrode was obtained from a Titrisol ampoule (Merck, Darmstadt, Germany). The precise amount of HCl in the Gd(III) solution was determined by the standard-addition method using 0.1 M HCl (Titrisol). The titrant solution used in the potentiometric titrations was obtained from a carbonate-free 0.1 M KOH commercial ampoule (Titrisol) and standardized by titration with potassium hydrogen phthalate, and rejected whenever the percentage of carbonate (Gran’s method) [39] was higher than 1% of the total amount of base. The potentiometric studies were performed with an automated potentiometric apparatus containing a Crison micropH 2002 millivoltimeter, a Crison microBu 2031 burette (Crison Instruments, Barcelona, Spain) and a Haake thermostatic bath (Haake Technik GmbH, Vreden, Germany, *T* = 25.0 ± 0.1 °C), controlled by the PASAT program. The glass and Ag/AgCl reference electrodes were previously conditioned in different DMSO/water mixtures of an increasing DMSO % composition (until 50%) and the response of the glass electrode was measured through the determination of the Nernst parameters by Gran’s method [39], obtained from strong acid–strong base (HCl/KOH) calibrations.

#### 3.1.2. Potentiometric Measurements

Potentiometric titrations of compound KEMPPr(3,4-HP)_3_, in the absence or presence of Gd(III), were performed in 50% (*w*/*w*) DMSO/water medium at an ionic strength (*I*) of 0.1 M KCl, *T* = 25.0 ± 0.1 °C, using 0.1 M KOH titrant. Each titration was repeated three times, for which the total volume was 20 mL, the total concentration of ligand (*C*_L_) was 3.2 × 10^−4^ M and the Gd^3+^/KEMPPr(3,4-HP)_3_ molar ratios were 0:1 and 1:1.

#### 3.1.3. Calculation of Equilibrium Constants

The stepwise protonation constants, *K*_i_ = [H_i_L]/[H_i−1_L][H] of the ligand, and the global stability constants of the Gd(III) complex, *β* (Gd_m_H_h_L) = [Gd_m_H_h_L_l_]/[Gd]^m^[H]^h^[L]^l^, were calculated by fitting the potentiometric data with the Hyperquad 2003 software [13]. The gadolinium hydrolysis model and ionic product of water (*K*_w_) were determined under the same experimental conditions (50% (*w*/*w*) DMSO/water medium, *I* = 0.1 M KCl, *T* = 25.0 ± 0.1 °C) and the values obtained for the stability constants (log *β* (GdH_−2_) = −15.17, log *β* (GdH_−3_) = −24.06) were included in the equilibrium model as well as the value of *K*_w_ (10^−14.95^). The species distribution curves were obtained with the Hyss 1998 program [13].

### 3.2. Molecular Modeling

Molecular modeling studies were performed to obtain insights on the molecular structure of the Gd(III) complex of KEMPPr(3,4-HP)_3_ and also to anticipate the most probable number of water molecules coordinated to gadolinium. The studies were achieved with the full geometry optimization of the gadolinium complexes by quantum mechanical calculations based on the density functional theory (DFT) methods included in the Gaussian 03 software [24,25], with the B3LYP hybrid functional [26] and the Stuttgart/Dresden (SDD) effective core potential (ECP) basis set [27,28]. No symmetry constraints were used during geometry optimization.

### 3.3. Biodistribution Studies

Animal studies were carried out in conformity with the national law and with the European Union (EU) Guidelines for Animal Care and Ethics in Animal Experimentation by researchers properly accredited by the national authorities. The animals were housed in a temperature- and humidity-controlled room with a 12 h light/dark schedule in animal house facilities approved by the Portuguese Authority of Food and Veterinary (DGAV) and maintained on a normal diet ad libitum.

The biodistribution of ^67^Ga-KEMPPr(3,4-HP)_3_ was evaluated in groups of three female CD-1 mice (randomly bred, Charles River) weighing approximately 25–28 g each. The animals were injected intravenously with 100 μL (5–10 MBq) of ^67^Ga-citrate via the tail vein and immediately after with 0.5 µmol of the ligand in 100 μL of saline solution. The mice were sacrificed by cervical dislocation at different time points (30 min, 1 h and 24 h) post-injection. The injected radioactive dose and the radioactivity remaining in the animal after sacrifice were measured using a dose calibrator (Capintec, Florham Park, NJ, USA). The difference between the radioactivity in the injected and sacrificed animal was assumed to be due to the total excretion from the whole animal body. Blood samples were taken by cardiac puncture at sacrifice. Then, tissue samples of the main organs were dissected, weighed and measured using a gamma counter (Berthold Technologies, Bad Wildbad, Germany). The biodistribution results were expressed as the percentage of the injected activity per organ (% I.A.).

## 4. Conclusions

Our goal was to investigate some of the relevant physic–chemical and biological properties of a new Gd (III) complex in view of the potential interest in this chelate as an MRI contrast probe. The hexadentate tripodal ligand (H_3_L, KEMPPr (3,4-HP)_3_), containing three 3,4-HOPO bidentate chelating moieties attached to a tri-carboxylic-cyclohexane-based backbone (KEMP acid), demonstrated a good capacity for wrapping and hexa-coordinating Gd (III), thus leaving free sites for extra coordination with water molecules. In particular, the results of solution equilibrium studies confirmed the formation of GdL complexes with a high thermodynamic stability (log *β* (GdL) = 26.59; pGd = 13.2 at pH = 6) under the used working conditions. The molecular simulation of the Gd complex indicated a slightly distorted octahedral coordination with one water molecule in the inner metal coordination, a relevant feature for the magnetic properties of this chelate. These results, together with the favorable in vivo stability and biodistribution profile of the complex, indicate the potential prospective interest in this kind of Gd chelate for future developments towards new MRI agents. Although further investigation is required through in vitro ^17^O NMR and NMRD studies to evaluate the actual number of water molecules in the inner coordination sphere and the proton relaxivity, we hope that the results presented herein may serve as a useful tool for medicinal chemists to design and create additional MRI contrast agents.

## Data Availability

The data are contained within the article and Supplementary Material.

## Supplementary Materials

The following supporting information can be downloaded at: https://www.mdpi.com/article/10.3390/molecules30061295/s1.

## References

[1] Wahsner J., Gale E.M., Rodríguez-Rodríguez A., Caravan P. (2019). Chemistry of MRI contrast agents: Current challenges and new frontiers. Chem. Rev..

[2] Aime S., Crich S.G., Gianolio E., Giovenzana G.B., Tei L., Terreno E. (2006). High sensitivity lanthanide(III) based probes for MR-medical imaging. Coord. Chem. Rev..

[3] Villaraza A.J.L., Bumb A., Brechbiel M.W. (2010). Macromolecules, dendrimers, and nanomaterials in magnetic resonance imaging: The Interplay between size, function, and pharmacokinetics. Chem. Rev..

[4] Cheng S., Abramova L., Gaab G., Turabelidze G., Patel P., Arduino M., Hess T., Kallen A., Jhung M. (2007). Nephrogenic fibrosing dermopathy associated with exposure to gadolinium-containing contrast agents—St. Louis, Missouri, 2002–2006. J. Am. Med. Assoc..

[5] Davies J., Siebenhandl-Wolff P., Tranquart F., Jones P., Evans P. (2022). Gadolinium: Pharmacokinetics and toxicity in humans and laboratory animals following contrast agent administration. Arch. Toxicol..

[6] Uzal-Varela R., Rodríguez-Rodríguez A., Wang H., Esteban-Gómez D., Brandariz I., Gale E.M., Caravan P., Platas-Iglesias C. (2022). Prediction of Gd(III) complex stability. Coord. Chem. Rev..

[7] Prybylski J.P., Semelka R.C., Jay M. (2017). The stability of gadolinium-based contrast agents in human serum: A re-analysis of literature data and association with clinical outcomes. Magn. Reson. Imag..

[8] Werner E.J., Avedano S., Botta M., Hay B.P., Moore E.G., Aime S., Raymond K.N. (2007). Highly soluble tris-hydroxypyridonate Gd(III) complexes with increased hydration number, fast water exchange, slow electronic relaxation, and high relaxivity. J. Am. Chem. Soc..

[9] Pierre V.C., Melchior M., Doble D.M.J., Raymond K.N. (2004). Toward optimized high relaxivity MRI agents: Thermodynamic selectivity of hydroxypyridonate /catecholate ligands. Inorg. Chem..

[10] Mendonça A.C., Martins A.F., Melchior A., Marques S.M., Chaves S., Villette S., Petoud S., Zanonato P.L., Tolazzi M., Bonnet C.S. (2013). New tris-3,4-HOPO lanthanide complexes as potential imaging probes. Complex stability and magnetic properties. Dalton Trans..

[11] Chaves S., Gwizdała K., Chand K., Gano L., Pallier A., Tóth É., Santos M.A. (2022). Gd^III^ and Ga^III^ complexes with a new tris-3,4-HOPO ligand, towards new potential imaging probes: Complex stability, magnetic properties and biodistribution. Dalton Trans..

[12] Grazina R., Gano L., Sebestik J., Santos M.A. (2009). New tripodal hydroxypyridinone based chelating agents for Fe(III), Al(III) and Ga(III): Synthesis, physico-chemical properties and bioevaluation. J. Inorg. Biochem..

[13] Gans P., Sabatini A., Vacca A. (1996). Investigation of equilibria in solution. Determination of equilibrium constants with the HYPERQUAD suite of programs. Talanta.

[14] Yang R., Schulman S.G. (2003). An operational pH in aqueous dimethylsulfoxide based upon the acidity dependence of the rate of a simple ionic recombination reaction in the lowest excited singlet state. Talanta.

[15] Chaves S., Marques S.M., Matos A.M.F., Nunes A., Gano L., Tuccinardi T., Martinelli A., Santos M.A. (2010). New tris(hydroxypyridinones) as iron and aluminium sequestering agents: Synthesis, complexation and in vivo studies. Chem. Eur. J..

[16] Pawlak Z., Bates R.G. (1975). Solute-solvent interactions in acid-base dissociation: Nine protonated nitrogen bases in water-DMSO solvents. J. Sol. Chem..

[17] Malla B., Neeraja R., Kumar J.S., Ramanaiah M. (2023). An eletrometric method for the determination of impact of DMSO-water mixtures on pKa values of salicylic acid derivatives. Int. J. App. Pharm..

[18] Kalidas C., Hefter G., Marcus Y. (2000). Gibbs energies of transfer of cations from water to mixed aqueous organic solvents. Chem. Rev..

[19] Beck M.T., Nagypal I. (1990). Chemistry of Complex Equilibria.

[20] Raymond K.N., Carrano C.J. (1979). Coordination chemistry and microbial iron transport. Acc. Chem. Res..

[21] Xu J., Franklin S.J., Whisenhunt D.W., Raymond K.N. (1995). Gadolinium complex of tris[(3-hydroxy-1-methyl-2-oxo-1,2-didehydropyridine-4-carboxamido)ethyl]- amine: A New Class of gadolinium magnetic resonance relaxation agents. J. Am. Chem. Soc..

[22] Puerta D.T., Botta M., Jocker C.J., Werner E.J., Avedano S., Raymond K.N., Cohen S.M. (2006). Tris(pyrone) chelates of Gd(III) as high solubility MRI-CA. J. Am. Chem. Soc..

[23] Werner E.J., Datta A., Jocher C.J., Raymond K.N. (2008). High-relaxivity MRI contrast agents: Where coordination chemistry meets medical imaging. Angew. Chem. Int. Ed..

[24] Parr R.G., Yang W. (1989). Density Functional Theory of Atoms and Molecules.

[25] Frisch M.J., Trucks G.W., Schlegel H.B., Scuseria G.E., Robb M.A., Cheeseman J.R., Montgomery J.A., Vreven T., Kudin K.N., Burant J.C. (2004). Gaussian 03, Revision C.02.

[26] Bauschlicher C.W. (1995). A comparison of the accuracy of different functionals. Chem. Phys. Lett..

[27] Haussermann U., Dolg M., Stoll H., Preuss H., Schwerdtfeger P., Pitzer R.M. (1993). Accuracy of energy-adjusted quasirelativistic ab initio pseudopotentials. Mol. Phys..

[28] Kuchle W., Dolg M., Stoll H., Preuss H. (1994). Energy-adjusted pseudopotentials for the actinides. Parameter sets and test calculations for thorium and thorium monoxide. J. Chem. Phys..

[29] Konings M.S., Dow W.C., Love D.B., Raymond K.N., Quay S.C., Rocklage S.M. (1990). Gadolinium complexation by a new DTPA-amide ligand. Amide oxygen coordination. Inorg. Chem..

[30] Junhu W., Masashi T., Takafumi K., Masuo T. (2007). Structure and bonding in some Gd(III) metal complexes studied by three-dimensional X-Ray analysis and ^155^Gd Mossbauer spectroscopy. J. Rare Earths.

[31] Lipinsky C.A., Lombardo F., Dominy B.W., Feeney P.J. (2001). Experimental and computational approaches to estimate solubility and permeability in drug discovery and development settings. Adv. Drug Deliv. Rev..

[32] Veber D.F., Johnson S.R., Cheng H.-Y., Smith B., Ward K.W., Kopple K.D. (2002). Molecular properties that influence the oral bioavailability of drug candidates. J. Med. Chem..

[33] Waring M.J. (2009). Defining optimum lipophilicity and molecular weight ranges for drug candidates—Molecular weight dependent lower log D limits based on permeability. Bioorg. Med. Chem. Lett..

[34] Bennion B.J., Be N.A., McNerney M.W., Lao V., Carlson E.M., Valdez C.A., Malfatti M.A., Enright H.A., Nguyen T.H., Lightstone F.C. (2017). Predicting a drug’s membrane permeability: A computational model validated with in vitro permeability assay data. J. Phys. Chem. B.

[35] Huttunen K.M., Raunio H., Rautio J. (2011). Prodrugs—From serendipity to rational design. Pharmacol. Rev..

[36] Pardridge W.M. (2012). Drug transport across the blood-brain barrier. J. Cereb. Blood Flow. Metab..

[37] Thompson M.K., Misselwitz B., Tso L., Doble D.M.J., Schmitt-Willich H., Raymond K.N. (2005). In vivo evaluation of gadolinium hydroxypyridonate chelates: Initial experience as contrast media in magnetic resonance imaging. J. Med. Chem..

[38] Le Fur M., Rotile N.J., Correcher C., Jordan V.C., Ross A.W., Catana C., Caravan P. (2020). Yttrium-86 is a positron emitting surrogate of gadolinium for noninvasive quantification of whole-body distribution of gadolinium-based contrast agents. Angew. Chem. Int. Ed..

[39] Rossotti F.J.C., Rossotti H. (1965). Potentiometric titrations using Gran plots: A textbook omission. J. Chem. Ed..

