# Supplementary material for: Gadolinium Complex with Tris-Hydroxypyridinone as an Input for New Imaging Probes: Thermodynamic Stability, Molecular Modeling and Biodistribution
Source: Molecules. 2025 Mar 13;30(6):1295. doi: 10.3390/molecules30061295 (PMC11945079; doi:10.3390/molecules30061295)
Supplement: Supplementary file 1 [file molecules-30-01295-s001.zip › molecules-3511561-supplementary.pdf]

Supplementary Material

Gadolinium complex with a tris-hydroxypyridinone as an input for new imaging probes: thermodynamic stability, molecular modeling and biodistribution

Inês Dias[a], Lurdes Gano[b], Sílvia Chaves[a*] and M. Amélia Santos[a*]

[a] Centro de Química Estrutural, Institute of Molecular Sciences, Departamento de Engenharia Química, Instituto Superior Técnico, Universidade de Lisboa, Av. Rovisco Pais 1, 1049-001, Lisboa, Portugal;

[b] Centro de Ciências e Tecnologias Nucleares, Instituto Superior Técnico, Universidade de Lisboa, CTN, Estrada Nacional 10 (km 139,7), LRS, Bobadela 2695–066, Portugal

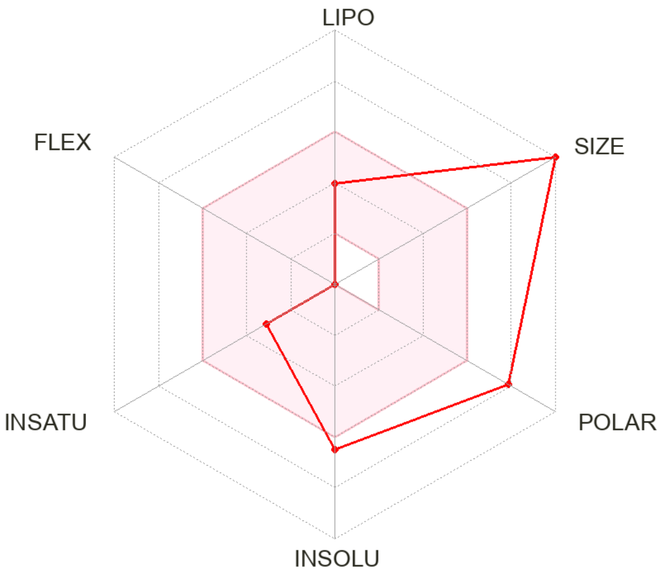

Figure S1. Schematic representation of some properties and pharmacokinetic parameters predicted by the swissADME software for the Gd-KEMPPr(3,4-HP)$_3$ complex, including in the colored zone the suitable physicochemical space for oral bioavailability. LIPO (lipophilicity): -0.7< Xlog P < +50; SIZE: 150 g/mol < MW < 500 g/mol; POLAR (polarity): 20 Å^2 < TPSA < 130 Å^2; INSOLU (insolubility): -6 < log *S* (ESOL) < 0; INSATU (insaturation): 0.25 < fraction Csp$_3$ < 1; FLEX (flexibility): 0 < Num. rotatable bonds < 9.
